# A prospective clinical study with one year follow up of deep caries management using a novel biomaterial

**DOI:** 10.1186/s13104-022-06041-z

**Published:** 2022-04-28

**Authors:** M. Roma, Ravi Gupta, Shreya Hegde

**Affiliations:** Department of Conservative Dentistry and Endodontics, Manipal College of Dental Sciences, Mangalore Affiliated to Manipal Academy of Higher Education, Manipal, Karnataka India

**Keywords:** Mineral trioxide aggregate, Portland cement, Pulp capping, Deep caries management

## Abstract

**Objectives:**

The objectives of this study was to check the outcome of the direct and indirect pulp capping procedure using MTA (Mineral Trioxide Aggregate) by comparing the pre—and post—operative pain by using VAS scale, associating the pre- and post- operative changes in intraoral periapical radiograph and clinical symptoms.

**Materials and methods:**

In this prospective clinical study 10 cases (5 for direct and 5 for indirect) with deep carious lesions (symptomatic) with no periapical changes were selected for the trial. The participants were subjected to deep caries management procedure under rubber dam where MTA is placed as pulp capping material followed by immediate restoration with sandwich technique using composite resin. The participants were followed up at recall visits of 1 month, 3 months, 6 months and 1 year intervals for clinical and radiographic evaluation.

**Results:**

The results of the study, analyzing the VAS, clinical symptoms and radiographic changes did not show any signs of pain, clinical and radiographic symptoms at 1 month, 3 months, 6 months and 1 year intervals.

**Conclusions:**

It was concluded that MTA can be used for deep caries management as a pulp capping material which being equivalent to calcium hydroxide.

## Introduction

Dental caries is one of the greatest challenges faced to the integrity of the tooth. Dental caries is a biofilm—mediated, sugar—driven, multifactorial disease, which occurs throughout life, in primary and permanents dentitions, damaging the tooth crown, as suggested by several and recent studies [1]. The dentist needs to acquire deeper understanding of the factors involved with caries management. To provide patients with best caries therapy and, risk assessment strategies, the, caries should be detection detected of caries at the earliest by using various advanced methods and techniques like, non-cavitated lesion management and conservative treatment modalities of management specific to defects, conservative caries removal and appropriate material selection. The basis for the current approach for caries removal in deep caries management is to understanding the changes that occur in tooth ultrastructure with the method and the rate of caries process progression is the basis for the current biological approach for caries removal especially in case deep caries management.

Traditional treatment for carious exposed pulps has been root canal treatment. To overcome this myth, deep caries management plays a significant role in persevering the vitality of the dental pulp. The main motto of operative therapy of deep carious lesions is the conservation of pulpal vitality of such teeth, which presents real task for dental clinicians. The complete caries excavation procedure is generally accepted by the clinicians over the stepwise excavation process for the treatment of deep caries management protocol [2–4]. In this treatment aspect, when the remaining dentinal thickness is less than 0.5 mm, the pulp is protected with a pulp protecting base. At present, there is substantial research done in dental materials focal point designed for the recognition of the dental materials which aid in production formation of strong dentinal bridge, other than long standing basic calcium hydroxide which is considered as gold standard material for pulp capping [5]. This is done basically to avoid uncontrolled necrotic zone created by calcium hydroxide [6, 7]. Calcium hydroxide is bound to have other disadvantages like disintegration in the oral fluids, [8] formation of tunnel defects in dentinal bridges which lead to destruction over a period of time [3, 4, 7, 8]. Numerous materials such as hydroxyapatite, zinc oxide eugenol, tricalcium phosphate, bioceramics, propolis, dentin adhesives have been tried on pulp. These specific components (e.g. hydroxyapatite, tricalcium phosphate) have been found to exert a pivotal role in several osteoconductive and osteoinductive process, as suggested by several and recent studies [9]. Tricalcium phosphate and its constituents like hydroxyapaptite have proven to enhance bone regeneration by escalating osteoconductivity for bone growth and improving bone remineralization by increasing the ion and growth factors release for osteoinductivity [10]. Recently introduced Mineral Trioxide Aggregate (MTA) is one of the successful materials for the therapeutic treatment of deep caries lesions. Mineral Trioxide Aggregate, better known as MTA was introduced by Mahmoud Torabinejad in 1993. ProRoot^®^ MTA (Dentsply Maillefer) a tricalcium based cement, has accentuated its usage in dentistry over the years in a successful way. Dentinal bridge formed with MTA when compared to that of calcium hydroxide, created effective hermetic seal which can unify with the dentin walls [11]. Literature search about MTA has shown various advantages like biocompatibility, [12] good sealing ability, [13] and being insoluble in oral fluids [14]. These beneficial effects have made it possible as a pulp capping agent for the treatment of deep caries management.

A lot of comparative studies has been conducted previously on treatment aspect of deep carious lesions, [15] nevertheless only a handful of them have studied the comparative effects of calcium hydroxide and MTA in the management of deep carious lesions [16]. Only few studies have studied the beneficial effects of MTA. This in vivo study discusses cases of clinical management of deep carious lesions using MTA in the institutional set up with a follow up over one year.

## Main text

### Materials and methods

This preliminary prospective longitudinal study was formulated and conducted on patients who attended the Department of Conservative dentistry and Endodontics. The methodology was accepted by the Institutional Ethical Committee (17061) of the University. Written informed consent was procured from all the patients who were involved in the study. Confidentiality pertaining to the information obtained during the course of the study was maintained at every stage of the study. This clinical trial was pre- registered in a Clinical Trials Registry (India) under the reference number REF/2019/05/025827.

### Selection of patients

Ten patients, aged 18–50 years were incorporated in this study. Symptomatic teeth with deep carious lesion having mild pain, mild tenderness and sensitivity to cold beverages and without any radiolucency or periapical changes in the intraoral periapical radiograph were included. Symptomatic cases with severe pain, swelling/extraoral swelling, intraoral sinus, with presence of periapical changes in radiograph and medical history such as diabetes mellitus, immune-deficient conditions were prohibited excluded from the study. An elaborate case history and a thorough clinical examination was carried out. Pre-operative IOPA radiograph in paralleling technique was taken for every case. The participants were introduced to deep caries management procedure under rubber dam. The case selection for direct pulp capping was done if there was exposure during excavation and if not, indirect pulp capping procedure was performed.

### Treatment protocol

#### Direct pulp capping procedure

The infected soft caries was excavated using sterile sharp spoon excavator. Bleeding was controlled with 2.5% NaOCl (Vishal Dentocare Pvt Ltd.) soaked sterile cotton plug. The disinfection of the cavity was done with 2% Chlorhexidine gluconate solution. MTA (MTA ANGELUS^®^) was mixed as per manufacturer’s instructions and was applied as pulp capping material followed by the placement of moist cotton and temporization was done. The patient was recalled after 24 h to remove the cotton pellet from the cavity and restored with glass ionomer (GC Glass Ionomer Universal Restorative) restoration.

#### Indirect pulp capping procedure

The infected soft caries was excavated using a sterile sharp spoon excavator leaving behind a layer of affected dentin. The disinfection of the cavity was done with 2% chlorehexidine gluconate (Vishal Dentocare Pvt Ltd.) solution. The disinfection of the cavity was carried out. MTA was applied as pulp capping material followed by the placement of moist cotton and temporization done. The patient was recalled after 24 h to remove the cotton pellet from the cavity and restored with glass ionomer restoration and composite.

**Fig. 1:**
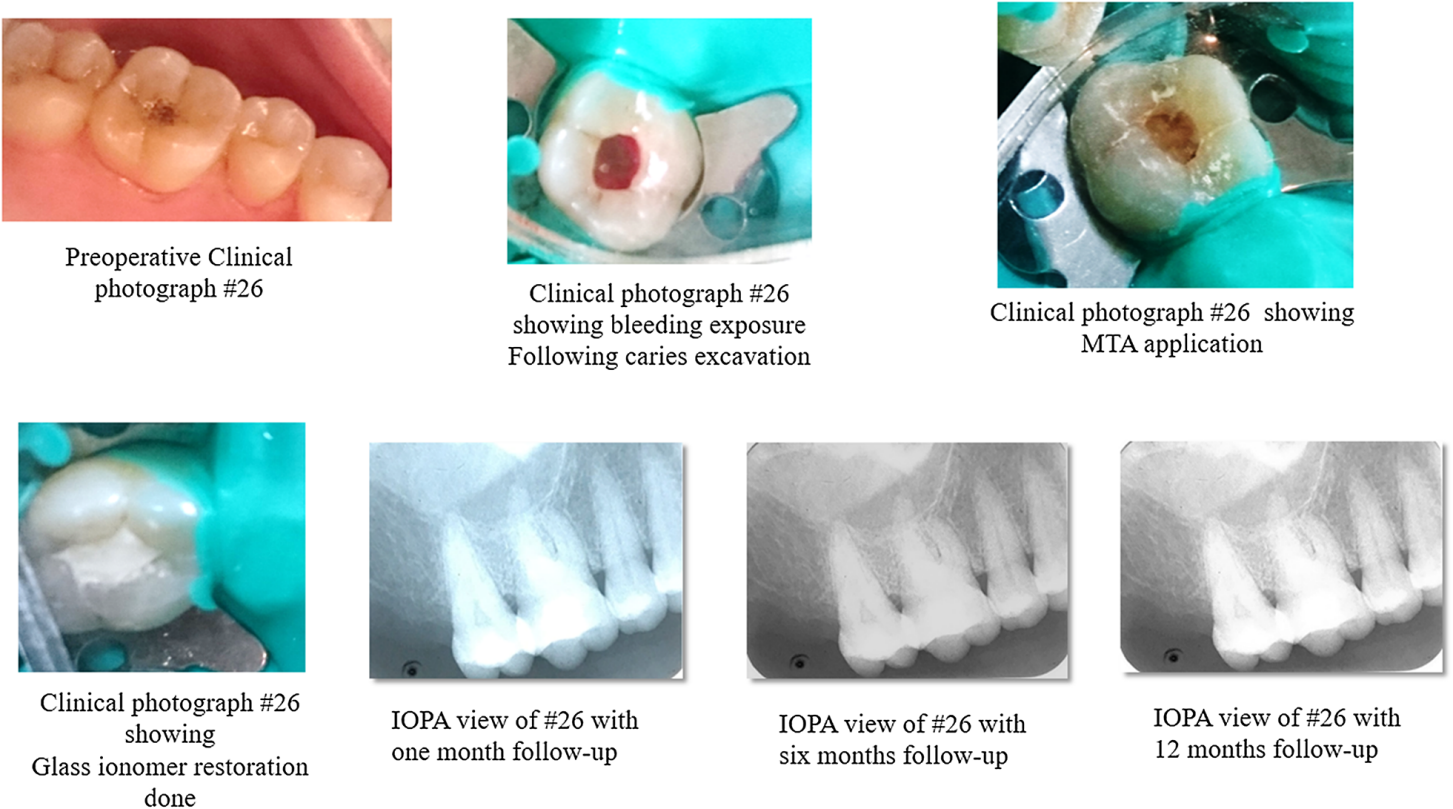
Clinical Procedure of MTA with respect to 26

**Fig. 2:**
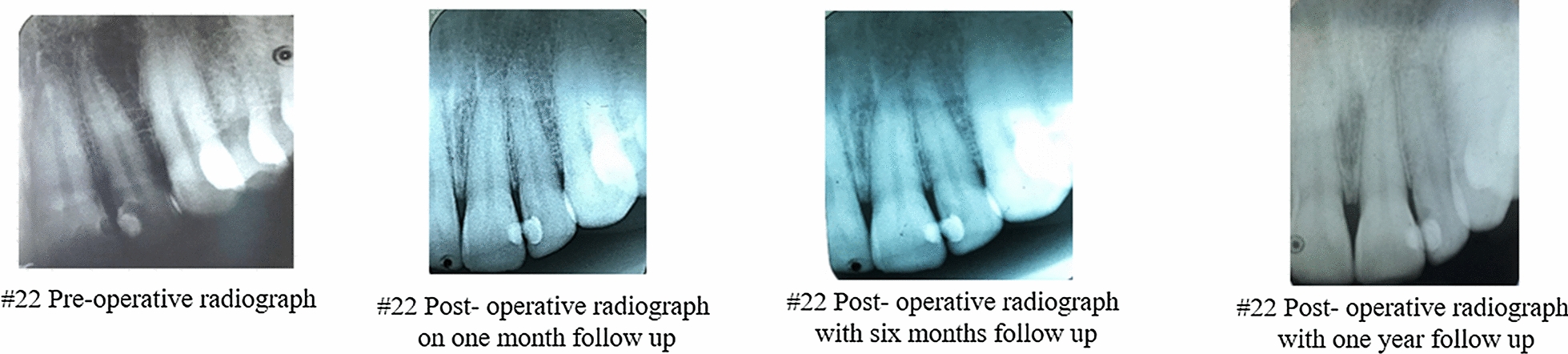
Radiographic outcome of MTA procedure with respect to #22 over a period of one year

The participants involved in the study was followed up at 1 month, 3 months, 6 months and one year intervals (Figs. 1 and 2). Visual Analogue Scale (VAS) was used for the assessment of the degree of pain in the scale of 0–10. Patients were assessed after a week. The cases were subjected to clinical examination to evaluate symptoms and post- operative IOPA radiographs at every interval for the radiographic changes. Radiographs was taken with paralleling technique and did not reveal any periapical changes.

### Statistical analysis

Data was analyzed using the Statistical Package for Social Sciences (SPSS), version 17 (SPSS Inc, Chicago IL). Descriptive statistics were tabulated. Chi square tests were applied.

## Results and discussion

### Results

This clinical trial consisted of 10 adult symptomatic permanent teeth with no periapical changes and rendered vital with pulp sensibility tests. All the 10 samples were subjected deep caries management procedure under proper isolation. The treated teeth did not have failures and the patients did not complain of any continuous pain or swelling. All the patients were recalled at follow‑up periods at 1 month, 3 months, 6 months and 12 months. All recall visits included both clinical and radiographic examinations. Clinically, there was total absenteeism of signs and symptoms and the teeth responded positively to pulp sensitivity tests, and radiographically, there was absence of periapical changes. The final treatment success rate was observed to be 100% (10 of 10) and the failure rate was 0.0% as shown in Table 1.

**Table 1:** Success outcome of deep caries management with MTA

Teeth analyzed (n = 10)	Direct pulp capping with MTA (n = 5)	Indirect pulp capping with MTA (n = 5)	Total
Success	5	5	10
Failure	0	0	0
P value	≥ 0.05	≥ 0.05	≥ 0.05

## Discussion

One of the biggest dilemmas in the treatment of deep caries lesions whether to restore it conservatively or to perform root canal treatment. In regard with this, there are two doctrine laid down which debated with the management of deep caries lesions. One doctrine debated that root canal treatment should be performed regardless of the pulpal status of the tooth. The second doctrine stated that the tooth can be treated conservatively with vital pulp therapy which helps in preservation of pulp [17].

Vital pulp is the key factor for vital pulp therapy procedure. Pulp sensibility tests do not accurately dictate the vital status of the pulp. Hence, the treatment success of deep caries management depends on preservation of the vital component of the pulp while removing the diseased one [18].

One more criterion for successful preservation of vital pulp is the placement of lining material. Over a decade, calcium hydroxide has been contemplated as the “gold standard” material for deep caries management procedure but due to certain disadvantages like tunnel defects, dissolution of material in oral fluids, etc. As an alternative to calcium hydroxide, the use of Mineral Trioxide Aggregate is in vogue. MTA being bioactive in nature incites the dental pulp cell proliferation, osteoblastic differentiation to form hard tissue in a shorter period of time and the dentinal interface had similar composition to hydroxyapatite [19]. MTA has been successfully used in various areas of endodontics as root end repair material, perforation repair, apexification etc. [20].

MTA is used in vital pulp therapy procedures and its outcome have shown tremendous successful results in direct and indirect pulp capping procedures thereby helping in dentinal regeneration and maintaining the pulpal vitality [11].

Khaled Wagih Al–Saudi et al. histologically studied the pulpal repair after direct pulp capping procedure with MTA and concluded the formation of complete dentinal bridge formation without any inflammatory pulpal response [21]. This is in accordance with the present clinical study which supports the fact that MTA is well used as pulp capping material.

The cases selected in this study were vital tooth without any history of spontaneous pain and periapical changes which played an important role for the success of the treatment Therefore, the condition of the pulp is very important for the conservative treatment modality. The degree of pulpal exposure and the pulpal bleeding highlights the severity of the pulpal inflammation [22]. For the direct pulp capping, various literature search has shown that “the amount of time to arrest the bleeding” should be between 5–10 min, and this point is taken into consideration to differentiate between reversible and irreversible pulpitis [23]. However, in this study the time to arrest the bleeding was within the confined time, which indicated vital pulp.

Optimum clinical conditions like (a) vital pulp, (b) biomimetic pulp capping material like MTA, (c) final restorative material play a very important role in the success of the treatment outcome. The success with this clinical study is mainly due to the nontoxic effect of Mineral Trioxide Aggregate on pulp and dentinal regeneration which will help in maintain the pulpal vitality and seals against all the microbes and the bacteria. In future, the clinical trial can be done with a larger study group.

## Conclusions

Mineral Trioxide Aggregate has proven to be a successful material in preservation of pulpal vitality in management of deep carious lesions. Biomimetic mineralization produced by MTA helps in formation of dentinal matrix has revolutionized its usage in the treatment of carious affected teeth in the field of dentistry.

## Limitation

Finally, the small number of patients included in this preliminary study meant that the analysis was underpowered. We recommend that future studies include a sufficiently large sample size to achieve adequate statistical power for analysis.

## Data Availability

The data used to support the findings of this study can be made available upon request to the corresponding author.

## Abbreviations

MTA: Mineral Trioxide Aggregate
IOPA: Intraoral periapical
